# The Role of MicroRNA in the Pathogenesis of Diabetic Nephropathy

**DOI:** 10.3390/ijms24076214

**Published:** 2023-03-25

**Authors:** Joanna Szostak, Anna Gorący, Damian Durys, Paweł Dec, Andrzej Modrzejewski, Andrzej Pawlik

**Affiliations:** 1 Department of Physiology, Pomeranian Medical University, 70-111 Szczecin, Poland; 2 Plastic and Reconstructive Surgery Department, 109 Military Hospital, 71-422 Szczecin, Poland; 3 Department of Surgery, Pomeranian Medical University, 71-422 Szczecin, Poland

**Keywords:** microRNA, diabetic nephropathy, diabetes mellitus, oxidative stress, transforming growth factor beta, biomarkers

## Abstract

Diabetic nephropathy is one of the most common and severe complications of diabetes mellitus, affecting one in every five patients suffering from diabetes. Despite extensive research, the exact pathogenesis of diabetic nephropathy is still unclear. Several factors and pathways are known to be involved in the development of the disease, such as reactive oxygen species or the activation of the renin–angiotensin–aldosterone system. The expression of those proteins might be extensively regulated by microRNA. Recent research suggests that in diabetic nephropathy patients, the profile of miRNA is significantly changed. In this review, we focus on the actions of miRNA in various pathways involved in the pathogenesis of diabetic nephropathy and the clinical usage of miRNAs as biomarkers and therapeutic targets.

## 1. Introduction

Diabetes mellitus (DM) is a great challenge for public health care worldwide as one of the leading causes of disability and premature death. From the 1980s, the number of adults affected by diabetes increased from 180 million to around 422 million in 2014 [1]. DM is characterized by severe micro- and macrovascular complications affecting numerous organs and systems [2,3,4]. Diabetic nephropathy (DN) is one of the most common and severe complications of DM, affecting around 20% of patients [5,6]. However, it is reported that up to 50% of chronic DM patients will develop end-stage DN and will require renal replacement therapy [7].

The disease develops gradually, with years-long latency episodes, and causes proteinuria and glomerulopathy [6]. Despite intense research in recent years, the exact pathogenesis of DN is still unclear. However, several factors and pathways are known to be involved in developing DN. Oxidative stress could be one of the main contributors to kidney failure, as reactive oxygen species (ROS) are common products of metabolic disorders involved in DM, such as hyperglycemia [8]. The ROS could directly impact the glomerulus, damaging the endothelial cells, mesangial cells, and podocytes [9,10]. Moreover, oxidative stress could also affect DN by activating other pathogenic pathways [11]. Renin–angiotensin–aldosterone system (RAAS) activation is involved in the development of kidney failure, mainly by altering intraglomerular hemodynamics and rearranging glomerulus structures [12]. Recently, the role of inflammation in the pathogenesis of DN was reported. The hemodynamic, metabolic, and biochemical disorders in DN can trigger a proinflammatory response in the kidney [13], increasing the production of cytokines such as interleukin 1 (IL-1) or IL-6 [14]. The leukocytes, monocytes, and macrophages attracted by cytokines infiltrate the kidney tissues and contribute to DN progression, directly damaging the glomerulus structures [15]. The expected result of oxidative stress, RAAS, and inflammatory response in the glomerulus is the hypertrophy of mesangial cells and the production of collagen by mesangial and epithelial cells, leading to kidney failure. However, it is crucial to establish specific molecular pathways involved in these mechanisms. Understanding signaling pathways could provide novel opportunities to diagnose and treat the disease. Currently, the pathways involved in inflammatory response and regulation of the cell cycle are considered to be associated with DN [6]. Those pathways include Janus kinase/signal transducers and activators of transcription (JAK-STAT), nuclear factor κB (NF-κB), transforming growth factor β1 (TGF-β1), mammalian target of rapamycin complex (mTOR) or phosphoinositide 3-kinase–protein kinase B (PI3K/Akt) [7,16,17,18,19].

MicroRNAs (miRNAs) are short-chained RNAs built of around 22 nucleotides [20]. In recent years, their function as posttranscriptional gene regulators was established, contributing to various biological processes, such as apoptosis, cell proliferation, or immune response [21]. Up to 5% of the human genome encodes the miRNAs [22], which regulate the expression of around one-third of protein-encoding genes [23]. miRNA sequences can be placed in targeted genes’ introns and originate from the primary transcript. However, numerous miRNAs are spread throughout the genome and are distant from targeted genes [24]. They are produced in a multistage process. The primary transcript—pri-miRNA—is cleaved in the nucleus and transformed into the cytoplasm as precursor (pre-miRNA). In the last stages, the pre-miRNAs are modified, and double strands unwind into separate single-stranded products. One forms the miRNA-induced silencing complex (miRISC) with Argonaute family proteins, while the second is degraded [25,26]. The miRISC is crucial in the posttranscriptional gene regulation process. It enables the bounding of the 5′ end of miRNA to complementary sites on 3′ mRNA, which restricts translation. Moreover, it also induces 5′-to-3′ degradation of mRNA by enhancing deadenylation and decapping of mRNA [27,28]. The repression of translation and downregulation of gene expression through mRNA degradation are two pathways of regulation mediated by miRNA [29]. It is known that alterations in miRNA levels are associated with numerous disease pathogeneses [30]. In DN, the profile of miRNA is significantly changed [7], highlighting the possible role of miRNA as a biomarker of the disease or target for novel therapies. In this review, we focus on the actions of miRNA in various pathways involved in DN pathogenesis and the clinical usage of miRNAs as biomarkers and therapeutic targets.

## 2. The Role of MicroRNA in the Pathogenesis of Diabetic Nephropathy

The pathogenesis of DN can be characterized by progressive hypertrophy and expansion of the glomerular mesangium, combined with accumulation of extracellular matrix (ECM) proteins and podocyte dysfunction [31]. This process is a result of combined effect of prolonged hyperglycemia, oxidative stress, inflammation, and advanced glycation end products [32]. Although it has not yet been exactly understood, the molecular basis of DN involves an intricate interplay between several key cell signaling pathways. Some of the most notable pathways include transforming growth factor beta (TGF-β), phosphoinositide 3-kinase–protein kinase B (PI3K/Akt), and collagen gene expression, the regulation of which, among others, we focus on in this review. It is important to distinguish that microRNAs contribute to the pathogenesis of DN in two manners—the upregulated miRs bind to the 3′UTR of renoprotective genes, which leads to their decreased expression, and the downregulated miRNA, in turn, can cause increased expression of genes involved in mesangial expansion or ECM accumulation [33]. Moreover, the landscape of miR expression differs to a significant degree in the early and late stages of DN. In early DN, dysregulated miRs mostly promote expression of ECM proteins, while in the later stages apoptosis and necrosis of tubular cells is observed [34].

Expression of miRNAs becomes deregulated under pathological conditions in chronic kidney diseases. A recent study has identified several aberrations of miRNA expression in renal fibrosis: upregulation of miR-142-3p, miR-223-3p, miR-21-5p, miR-142-5p, and miR-214-3p and downregulation of two miRs—miR-29c-3p and miR-200a-3p [35]. The general consensus in the field is that with the progression of kidney disease and decrease in glomerular filtration rate, serum levels of miRs decrease. Total levels of five specific miRs have been found to be reduced in advanced kidney failure (miR-16, miR-21, miR-155, miR-210, miR-638) [36]. Moreover, in advanced stages of chronic kidney disease, levels of miR-125b, miR-145, miR-155 decrease [37].

Aside from DN, modulation of miRNA levels could yield potential therapeutic benefit in hypertensive nephropathy. Knockdown of miR-103a-3p resulted in decreased angiotensin II-induced effects on the kidney [38]. This effect was mediated by SNRK, whose expression is downregulated by miR-103a-3p. It is important to underline the fact that the effect of miR modulation was investigated during the development of chronic kidney disease, rather than studying the therapeutic benefits in already established disease. Nevertheless, the results suggest potential therapeutic benefit of miR modulation.

### 2.1. miRs Involved in Modulating TGF-β Signaling

Experimental data suggest that TGF-β is involved in ECM accumulation in the mesangium, causing glomerular hypertrophy and leading to renal damage [39]. Additionally, the accumulation of ECM then causes fibrosis and scarring [40]. Due to its profibrotic action, high levels of TGF-β are often found in both glomerular cells and tubular cells in advanced DN [41,42].

Prolonged serum hyperglycemia, which is a staple of long-standing DM, increases the expression of TGF-β. This effect is mediated through protein kinase C, polyol, and hexosamine pathways [43,44]. High glucose induces upstream stimulatory and transcription factors of TGF-β, such as upstream transcription factors 1 and 2 [45]. TGF-β1 induces collagen expression and its expression is regulated by E-box repressor Zeb1/2. miR-192 both upregulates TGF-β1 and negatively regulates Zeb1/2, resulting in further relief of repression [46]. Additionally, miR-192 targets the expression of Smad-interacting protein 1 (SIP1), which is another E-box repressor [47]. Similarly, miR-200b/c both inhibit Zeb1, affecting TGF-β1 autoregulation, in turn increasing COL1α2 and COL4α1 expression [46]. Another effect of miR-200b/c could be TGF-β Akt activation through FOG2 (zinc finger protein ZFPM2) downregulation. Through this mechanism, miR-200b/c can also activate ERK through PI3K, leading to mesangial hypertrophy [48]. ECM accumulation is also believed to be driven by miR-1207-5p, which is abundantly expressed in DN kidney cells. It is upregulated by TGF-β1 and high glucose, and increases the expression of TGF-β1 by reducing the expression of PMEPA1 (prostate transmembrane protein, androgen-induced 1), PDPK1 (3-phosphoinositide-dependent protein kinase 1), and SMAD7 (SMAD family member 7) [49].

miR-21 has been identified as involved in human kidney fibrosis, and it has been demonstrated that TGF-β increases miR-21 via a Smad3-dependent mechanism [50]. Moreover, miR-21 likely induces renal injury through targeting Smad7, as its knockdown restores Smad7 levels, leading to suppression of TGF-β and NF-κB signaling [51]. TGF-β1 is also implicated in glucose-induced phenotypic transition of mesangial cells. miR-215 in this process targets catenin-beta interacting protein 1 (CTNNBIP1), in turn leading to β-catenin activation and α-SMA and fibronectin expression [52].

TGF-β1 and TGF-β2 are also believed to downregulate several downstream feedback regulators of their expression, such as miR-200a and miR-141. Under normal conditions, both miRs serve a protective role through downregulating Smad-3 activity, preventing TGF-β–dependent EMT [53]. Advanced glycation end products, under prolonged diabetic conditions, cause significant miR-29b downregulation in a mechanism that is mediated by TGF-β and Smad3. Loss of miR-29b results in further TGF-β/Smad3-mediated renal fibrosis, microalbuminuria, inflammation, and immune injury [54].

### 2.2. miR Affecting Collagen Expression

Accumulation of the ECM proteins collagen and fibronectin (FN) results in thickening of the glomerular membrane and mesangial matrix expansion, both of which are characteristic of DN [55]. The expression of miR-21 correlates strongly with collagen IV (ColIV), and FN. The data suggest that this effect is mediated by MMP9/TIMP1 [56].

miR-377 is believed to indirectly increase FN production and it is abundantly expressed when mesangial cells are exposed to high glucose. Its overexpression is associated with reduced expressions of p21-activated kinase and superoxide dismutase, which in turn enhances FN production [57].

The miR-29 family is known to contribute to DN directly through modulating multiple molecular targets, such as PI3K, DNA methyltransferase, or collagen. Experimental data suggest that miR-29c targets Spry1 under hyperglycemic conditions, interacting with its 3′-UTR [58]. Spry1 is a known angiogenesis inhibitor, whose silencing alters interactions between cells and ECM proteins [59]. In hyperglycemic conditions, miR-29c not only promotes FN synthesis but also exhibits potent proapoptotic effects [58].

Profibrotic cytokine TGF-β1 regulates the expression of select microRNAs, including the miR-29 family. Experimental data suggest that TGF-β1 inhibits the expression of miR-29, promoting expression of ECM elements [60]. Either high glucose or TGF-β1 significantly downregulate miR-29a, which, when overexpressed, causes negative regulation of collagen IV in proximal tubule cells by targeting the 3′UTRs of *col*4*a*1 and *col*4*a*2 genes [61].

Another miR that indirectly affects renal fibrosis is miR-135a. It promotes cell proliferation and increases FN and collagen I synthesis by regulating the transient receptor potential cation channel, subfamily C, member 1 (TRPC1) in mesangial cells [62]. TRPC1 activates calcium entry into multiple tissue types, including human nephrons [63]. Its expression has been found to be reduced in kidneys from patients with DN [64].

### 2.3. miR Regulating the PTEN-Dependent Pathways

Phosphatase and tensin homologue (PTEN) is a tumor suppressor. In addition to dephosphorylation of phosphatidylinositol (3,4,5)-triphosphate (PIP3), it is a vital negative regulator of the proto-oncogenic phosphatidylinositol 3-kinase (PI3K)–protein kinase B (Akt) and mammalian target of rapamycin (mTOR) pathways [65]. Recent research suggests that it plays a role in regulating renal fibrosis and epithelial–mesenchymal transition [66]. Emerging studies suggest that hyperglycemia stimulates PI3K and inhibits PTEN, in turn activating the Akt/mTOR pathway and initiating DN via glomerular basement membrane thickening and expansion of the mesangial matrix [67].

The expression of PTEN is influenced by several miRs. TGF-β was identified to upregulate both miR-216a and miR-217, which then activate Akt signaling by targeting PTEN in glomerular mesangial cells [68,69]. miR-192 contributes to this process as an upstream regulator. Akt activation in this miR circuit affects ECM gene expression, cell hypertrophy and survival [69].

In renal cells, high glucose or TGF-β upregulates the expression of miR-21, which then affects PTEN expression and phosphorylation of Akt. Additionally, proline-rich AKT substrate of 40 kDa (PRAS40) becomes inactivated, thereby increasing target of rapamycin complex 1 kinase (TORC1) activity, which results in renal cell hypertrophy and FN expression [70]. In another study, miR-21 levels correlated with tubulointerstitial fibrosis and estimated glomerular filtration rate. Furthermore, miR-21 was induced by TGF-β and further enhanced the downregulation of PTEN. The elicited phosphorylation of Akt induced was greater than when induced by TGF-β1 alone. Additionally, miR-21 reduced the level of SMAD7 and prevented its increase by TGF-β. On the contrary, SMAD3 phosphorylation was increased [71]. Overexpression of miR-214 decreased the levels of PTEN and increased Akt activity in a manner similar to high glucose, leading to phosphorylation of PRAS40 and tuberin. Furthermore, miR-214 controlled mTORC1 activity, leading to cell hypertrophy and expression of the matrix protein FN [72].

**Figure 1:**
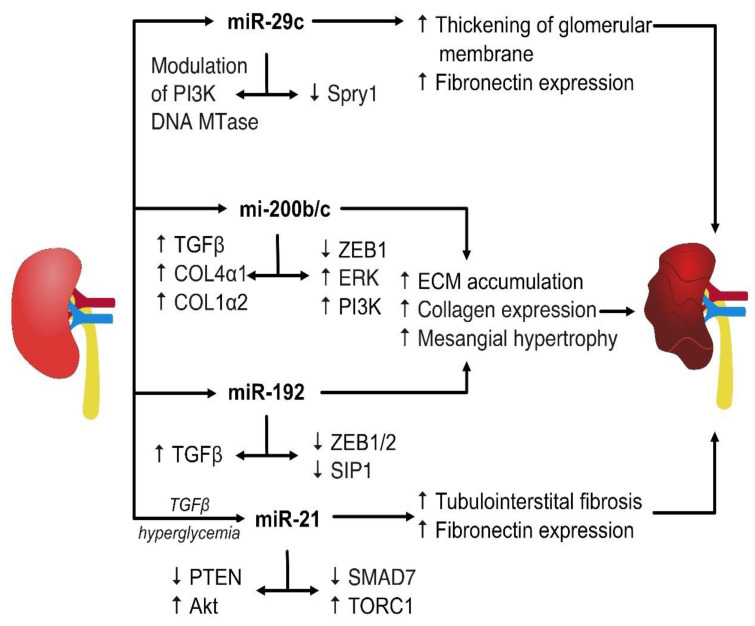
The miRs are involved in numerous molecular pathways contributing to DN. The most common involve upregulation of TGF-β1 and collagen synthesis or downregulation of PTEN. TGF-β1—transforming growth factor β1; PI3K/Akt—phosphoinositide 3-kinase–protein kinase B; DNA MTase—DNA methyltransferase; Spry1—Protein sprouty homologue; ERK—extracellular signal-regulated kinase; SIP1—Smad-interacting protein 1; PTEN—phosphatase and tensin homologue; SMAD7—SMAD family member 7, TORC1—target of rapamycin complex 1 kinase.

Studies suggest that miR-22 is associated with autophagy in DN by suppressing autophagic flux in renal tubular cells, partially by targeting PTEN, which leads to induction of Col IV and α-SMA [73]. Similarly, the autophagic flux was inhibited by miR-141-3p, again by the means of PTEN/Akt/mTOR pathway upregulation by high glucose [74]. Microarray analysis studies have revealed that miR-25 is significantly downregulated in renal tissue in DN patients. Its low expression is coupled with production of reactive oxygen species, cell apoptosis induced by cell damage, and increased activity of cleaved caspase 3. Further studies demonstrated that PTEN was a direct functional target of miR-25 [75]. A short summary of main miRs’ impact on molecular gene activities involved in the pathogenesis of DN is presented in Figure 1.

### 2.4. miR Affecting Other Pathways Involved in the Pathogenesis of the Disease

Several other miRs are involved in the pathogenesis of DN, via targeting several pathways. For example, miR-21 targets the 3′ UTR of the tissue inhibitor of metalloproteinase 3 (TIMP3) gene, decreasing its expression in glomerular cells [76]. TIMP3 is known for controlling inflammation and fibrosis, and its decrease contributes to DN by increasing the activation of matrix metalloproteinase-2 (MMP-2) and oxidative stress [77].

Apoptosis of glomerular cells is one of the characteristic events in the pathogenesis of DN. Normally, the balance between pro- and antiapoptotic factors is maintained in glomerular cells. However, in high glucose, miR-195 reduced the levels of antiapoptotic BCL2 (B-cell lymphoma 2), contributing to apoptosis via an increase in caspase 3 [78]. Moreover, miR-218 accelerated cell apoptosis directly by suppressing heme oxygenase 1 (HO-1), which is normally upregulated in renal injury [79].

A summary of the effect of these miRs, among many others [80,81,82,83,84,85,86,87,88,89,90,91], can be found in Table 1.

## 3. miRs That Elicit Protective Properties in Diabetic Nephropathy

By referring to the complementary segment of mRNA, miRNA inhibits its expression and translation of proteins. In some cases, the molecular block degrades the mRNA transcript at the time of dsRNA formation. It is possible that the miRNA complex may blocks the protein translation process without causing mRNA degradation [93]. Some siRNA and miRNA therapies have been approved for clinical trials and have focused, for example, on the treatment of diabetes or hepatitis and fatty liver disease [94].

Studies have shown an immunomodulatory connection between miRNAs and various complications of diabetes, including the pathogenesis of DN. It is an important regulator. The role of in vivo miRNA-146a in DN has been investigated in animals and is a known example of an anti-inflammatory miRNA. The results suggested that in early DN, miRNA-146a shows stronger expression and anti-inflammatory and renoprotective roles [95]. A similar medical study has proven that modified miRNA-146a from umbilical cord-derived mesenchymal stem cells has increased anti-inflammatory efficiency and improved kidney function in rats with DN, which had been induced by streptozotocin. The role of miRNA-146a was connected to macrophage polarization as enhanced protection against kidney damage [96]. Additional research proposed molecular mechanisms by using urinary-derived human stem cells. Exosome of human urine-derived stem cells overexpressing miRNA-16-5p may inhibit vascular endothelial growth factor A. It may also suppress apoptosis and promote podocyte proliferation. This will alleviate the consequences of podocyte damage in diabetic nephropathy [97]. Another animal study has shown concentration of some factors, such as TGF-β2 or NADPH oxidase subunit 4 (NOX4), in DN. Their accumulation in the matrix causes renal fibrosis and oxidative stress. These key factors are closely related to regulation by miRNA-25b molecules that reduced their concentration. In the results of one study, miRNA-25b level was reduced in diabetic rats, as well as in mesangial cells, treated with high concentration of a glucose substance. This was accompanied by an increase in NOX4 expression [82]. Finally, miRNA-29b showed a protective role in another study by inhibiting the TGF-β/SMAD3 pathway of kidney inflammation. The collected data allowed the conclusion that miRNA-25 and miRNA-29b may protect against development of DN by inhibiting TGF-β2 and NOX 4 synthesis, respectively [54].

## 4. miRs as Therapeutic Targets in Treatment and Prevention of DN

miRNAs are associated with development of diabetes complications and can be biomarkers to control the progression of the disease [94,98]. Patients with diabetes are characterized by lower levels of miR021, miR-27a, and miR-146a in sciatic nerve tissue [98,99]. Also noteworthy is that neuropathy associated with diabetes is more likely to occur among patients with type 2 diabetes (T2D) who have a polymorphism in the miR-146a, miR-27a, and miR-124a genes [99]. Research shows that injection of the exosomal miRNAs (miR-28, miR-31a, miR-130a) isolated from Schwann cells, which were exposed to high glucose levels, may significantly increase the risk of diabetic peripheral neuropathy (DPN) occurrence in diabetic leptin receptor-deficient (db/db) mice [98,100,101]. Furthermore, exosomal miRNAs can also be a therapeutic target in treatment of diabetes complications, such as DPN [98,102]. Exosomes used in therapy are cellular nanovesicles responsible for intercellular transportation, which makes them eligible drug carriers [103]. One study proved that administration of those exosomal miRNAs from healthy Schwann cells (SC-Exo miRs) can lessen DPN progression in db/db mice with T2D [99]. Therapeutic effects of exosomal miRNAs may be associated with the increased levels of intraepidermal nerve fiber (IENF), owing to the fact that IENF depletion is a valid first indication in DPN [99]. Moreover, neuropathic symptoms of DPN can be decreased by treatment with SC-Exos, due to their role in reducing axonal and myelin impairment and to the fact that they have a positive impact on nerve conduction velocities (NCV) [99]. Another study indicated the important role of mesenchymal stromal cell (MSC)-derived exosomes in the treatment of DPN in diabetic mice [104]. MSC exosome administration leads to a lowered threshold for thermal and mechanical stimuli and enhanced nerve conduction velocity [104]. They also have a significant impact on myelin thickness and axonal diameter increase of sciatic nerves [104]. Likewise, SC-Exos, the MSC exosomes, are responsible for IENF growth [104]. Furthermore, MSC exosomes have an influence on proinflammatory cytokines that play a role in inflammatory reaction suppression [102]. The study showed that Let-7a, miR-23a, and miR-125b are associated with macrophage polarization adjustment by targeting the TLR4/NF-kB signaling pathway [102], thus ameliorating neurovascular function [104].

In conclusion, there is still a lot to be discovered in the exosomal miRNA-dependent treatment of DPN, but it is evidently a very important and promising way of dealing with this particular diabetes complication. Due to their properties, SC-Exo miRs, as well as MSC exosomes, may have significant value for diabetes patients.

## 5. Select miR as Biomarkers of the Disease

Disease biomarkers are molecules that can be used at different stages of diagnosis, therapy, or prognosis evaluation. A proper biomarker should be sensitive, specific, stable, and easily acquired in a noninvasive way. According to conducted studies, extracellular miRNAs fulfill these features [105]. They are considered to act as epigenetic biomarkers in various diseases [106]. By utilizing uncomplicated and sensitive methods, they can be obtained from blood and urine, as well as other body fluids, even after lengthy storage [107]. Serum miRNAs are suggested to be stable in blood despite high ribonuclease activity in plasma, due to their containment in exosomes and binding to Argonaut proteins and HDL [108]. Furthermore, their expression is tissue-specific [109]. For instance, some miRNAs have been explicitly associated with the kidney, such as miR-192, miR-194, miR-204, miR-215, and miR-216 [110]. Fluctuations in circulating miRNA levels in biological materials have been reported in various conditions, such as cardiovascular diseases, neurodegenerative disorders, cancer, and T2D, including diabetic kidney disease (DKD) [105]. However, the involvement of miRNA in several diseases remains a problem. DKD is one of the most prevalent diabetic complications causing reduced life expectancy. Therefore, the search for predictive biomarkers for early diagnosis, advanced DKD, and end-stage renal disease has drawn a lot of attention and investment in recent years [111]. Some miRNAs as biomarkers in renal diseases have already been acknowledged and their usefulness proved in monitoring the course of the disease, as well as determining the accuracy of the diagnosis or treatment efficacy [112].

Multiple studies have evaluated serum and urinary miRNA levels in patients suffering from type 1 and type 2 diabetes in different DKD stages [113,114,115,116]. However, these studies enrolled a small number of patients [113] and described only cross-sectional associations between miRNAs in urine and albuminuria [113,114,115]. None of the reported miRNAs coincided as being relevant in DKD, indicating there is as yet no convincing proof for the clinical implementation of miRNAs in the prediction of DKD progression [117].

On the other hand, many differentially expressed miRNAs were shown in the urinary exosome microarray in patients with T2D and DKD. Delić et al., in their study on miR-320c and miR-6068, upregulated miRNAs in urinary exosomes of DKD patients, reported that dysregulation of urinary exosomal miRNAs was associated with microalbuminuria in DKD patients, but not in DM patients [113]. Similarly, Zhao et al. confirmed that the expression of urinary exosomal miRNA in DKD patients was changed. Furthermore, they implied the involvement of miR-4534 in the early formation of microalbuminuria. In conclusion, miR-4534 might be expected to become a new biomarker for the progression of T2D DKD disease [118]. It has also been shown that some miRNAs, such as miR-25, miR-93, miR-200, and miR-451, are downregulated in DKD [119]. Accordingly, both up- and downregulation of DKD-suppressing and DKD-promoting miRNAs can be used as possible therapeutic targets in DKD [120]. However, there are still some contradictory results regarding the levels of miRNA expression in disease models. Hong et al. showed that miR-193a-3p could be used to distinguish patients with DKD. Their analysis revealed that the high expression of miR-193a-3p significantly reduced the dialysis-free survival of DKD patients. The involvement of miR-193a-3p in DKD pathogenesis may suggest that it can act as a new diagnostic biomarker as well [121]. There is no persuasive evidence to prove the diagnostic value of miRNAs in the prediction of DKD progression. Further prospective or randomized controlled trials should be conducted for clinical validation. In conclusion, miRNAs show a lot of promise as novel biomarker candidates for DKD and should be evaluated in future studies.

## 6. Conclusions

DN, a significant and common complication of type 1 and type 2 diabetes, in its primary stages has no specific symptoms. It is diagnosed based on proteinuria and a drop in glomerular filtration rate. Glomerular dysfunction is followed by end-stage renal disease. DN is a significant financial burden for health care in developed countries because it requires dialysis lasting years or costly immunosuppressive treatment after kidney transplantation. Despite numerous attempts to improve the situation of patients, there is still neither efficacious prevention nor treatment for DN. Therefore, a complete understanding of DN pathogenesis is a crucial step to the development of less problematic and more efficient therapy.

In this systematic review of the most important and novel studies, we summarized progress in research on the role of miRNA in DN. Scientists have tried to understand the importance of miRNA as a crucial connection between various inherited or acquired risk factors, such as the presence of advanced glycation end products (AGEs) or oxidative stress, and the development of DN. The miRNA profile is often changed during diseases, including DN. Moreover, various miRNAs appear to be associated with hyperglycemia and insulin resistance. Other miRNAs are explicitly associated with kidneys or are present in urinary exosome and connected with DN progression. Moreover, hypertension and hyperfiltration, common amongst hyperglycemic patients, seem to depend on miRNA aberrations.

In our review, we described the role of miRNA in the regulation of collagen expression in tubular epithelium and mesangial cells during mesangial expansion. We summarized investigations on the hypothetical connection between miRNA involvement in signaling pathways within renal cells and the pathogenesis of DN. Still, improving methods of miRNA profiling lets researchers study miRNA transcripts as potential biomarkers or therapeutic targets. Mammalian target of rapamycin complex (mTOR), transforming growth factor β (TGF-β), and phosphoinositide 3-kinase–protein kinase B (PI3K/Akt) expression is particularly important during the development of DN. These emerging miRNA-based mechanisms might one day serve as a potent target in DN treatment.

An increasing number of dissertations support a vital role of miRNAs in the progression of DN. There are pieces of evidence suggesting that miRNA-derived interventions are critical to the effective treatment of DN. However, considerably more studies are required to refine the clinical management of DN. Despite efforts to find a crucial target for therapeutic intervention in DN, the search for the perfect biomarker is ongoing. We might expect first attempts to translate findings into better therapeutic approaches for the proper treatment depending on research progress.

## Figures and Tables

**Table 1:** Summarizing the effect of select microRNA on the pathogenesis of diabetic nephropathy with regard to target protein, type of regulation, outcome, and model used in the study.

miRNA	Up/Downregulation	Target Protein or Pathway	Outcome	Model	Reference
miR-192	Up	ZEB1/SIP1	TGF- β -induced increased COL1α1 and COL1α2 expression	Mouse	[47]
miR-192	Up	ZEB1/2	Increased TGF-β signaling, increased fibrosis	Mouse	[46]
miR-216a	Up	PTEN, YBX1	TGF-β-induced collagen expression	Mouse mesangial cells	[68]
miR-217	Up	PTEN	Increases mesangial cell survival	Mouse mesangial cells	[69]
miR-200b/c	Up	ZEB1	Mediates TGF-β1 autoregulation, increases COL1α2 and COL4α1 expression	Mouse	[46]
miR-200b/c	Up	FOG2, TGF-β	Akt kinase activation, glomerular mesangial hypertrophy	Mouse	[48]
miR-21	Up	PTEN, PRAS40, TORC1	Renal hypertrophy through Akt/TORC1 pathway	Human mesangial cells	[70]
miR-21	Up	SMAD7	activation of the TGF-β and NF-κB signaling pathways	Mouse	[51]
miR-21	Up	SirT1	TIMP3 downregulation	Mouse	[76]
miR-21	Up	MMP9/TIMP1	Increased collagen and FN expression	Mouse	[56]
miR-21	Up	PTEN-SMAD7	Regulation of renal tubular ECM via Smad3/Akt pathway	Mouse	[71]
miR-377	Up	PAK1, SOD	Increased FN production	Mouse	[57]
miR-195	Up	Bcl2	Promotes apoptosis via enhanced caspase cascade	Mouse	[78]
miR-215	Up	CTNNBIP1	Promotes TGF-β driven mesangial cell phenotypic transition	Mesangial cells	[52]
miR-124	Up	Integrin α3β1	Downregulation of podocyte α3 and β1 that leads to adhesion damage under mechanical stress	Human podocytes	[84]
miR-29c	Up	Spry1	Activation of Rho kinase, albuminuria, mesangial matrix accumulation	Mouse podocytes	[58]
miR-29c	Up	Tristetrapolin	Promotes inflammatory response via increase of TNF-α and IL-6	Human plasma and urine	[80]
miR-1207-5p	Up	PMEPA1, G6PD, PDPK1	ECM accumulation through TGF-β	Human podocytes	[49]
miR-135a	Up	TRPC1	Increases FN and collagen synthesis	Human serum and kidney tissue, mouse	[62]
miR-200a	Down	TGF-β2	Increased FN and collagen synthesis	Rat tubular epithelial cells	[53]
miR-141	Down	TGF-β2	Increased FN and collagen synthesis	Rat tubular epithelial cells	[53]
miR-29	Down	Collagen I and collagen IV	Increased collagen production and fibrosis	Rat tubular epithelial cells, mesangial cells, podocytes	[60]
miR-29a	Down	COL4α1, COL4α2	Excess collagen deposition	Human tubular cel line	[61]
miR-29a	Down	HDAC4	Podocyte protein deacetylation and degradation as well as renal dysfunction	Mouse	[85]
miR-29b	Down	TGF-β/Smad3	Upregulation of collagen matrix in mesangial cells	Mouse	[54]
miR-451	Down	YWHAZ	Upregulation of p38 MAPK signaling, mesangial proliferation	Mouse mesangial cells	[81]
miR-25	Down	NOX4	Increased NOX4 expression	Rat	[82]
miR-93	Down	VEGF-A	Increased fibrosis	Mouse, endothelial cells, podocytes	[86]
miR-22	Up	PTEN	Suppression of autophagic flux and induction of Col IV and α-SMA expression	Rat renal cells	[73]
miR-27a	Up	PPARγ	Increased ECM accumulation, proteinuria	Rat	[87]
miR-92b-3p	Up	SMAD7	Increased TGF-β signaling and renal fibrosis	Rat	[83]
miR-141-3p	Up	PTEN	Increased renal fibrosis through miR-141-3p/PTEN/Akt/mTOR pathway	Rat	[74]
miR-181a	Up	Deptor	Leads to TGFβ-induced mesangial cell hypertrophy and matrix protein FN expression	Rat mesangial cells	[88]
miR-184	Up	LPP3	Tubulointerstitial fibrosis and albuminuria	Rat	[89]
miR-214	Up	PTEN	Cell hypertrophy and expression of the matrix protein FN	Mouse	[72]
miR-218	Up	HO-1	Acceleration of podocyte apoptosis through directly downregulating HO-1 and facilitating p38-MAPK activation	Mouse podocytes	[79]
miR-23c	Down	MALAT1	Contributes to hyperglycemia-induced cell pyroptosis	Renal tubular epithelial cells	[90]
miR-25	Down	PTEN/Akt	Oxidative stress and apoptosis in renal tubular epithelial cells	Renal biopsy tissue	[75]
miR-30c	Down	CTGF	Upregulation of CTFG results in renal fibrosis	Human kidney tubular epithelial cells	[91]
miR-130b	Down	Snail	Deregulated E-CADHERIN, VIMENTIN, COLLAGEN IV and α-smooth muscle actin (α-SMA), key mediators of thus affecting epithelial-to-mesenchymal transition	Rat	[92]

ECM—extracellular matrix, FN—fibronectin, NF-κB—nuclear factor κB, TGF-β—transforming growth factor β.

## Data Availability

Not applicable.
